# Masseter-to-facial nerve transfer for facial nerve reanimation

**DOI:** 10.3171/2022.9.FOCVID22107

**Published:** 2023-01-01

**Authors:** Hussam Abou-Al-Shaar, Michael Karsy, Ilyas M. Eli, Jayant P. Agarwal, Barbu Gociman, Mark A. Mahan

**Affiliations:** 1 Department of Neurological Surgery, University of Pittsburgh Medical Center, Pittsburgh, Pennsylvania;; 2 Department of Neurosurgery, Clinical Neurosciences Center, University of Utah, Salt Lake City, Utah;; 3 Department of Neurosurgery, Ascension St. Thomas Rutherford Hospital, Murfreesboro, Tennessee; and; 4 Division of Plastic Surgery, University of Utah, Salt Lake City, Utah

**Keywords:** facial nerve, reanimation, nerve transfer, pediatric, video

## Abstract

Smooth symmetric facial muscle function is important for social interactions. When lesions of the facial nerve occur, achieving complete restoration of balanced and spontaneous facial function can be challenging. In this video, the authors demonstrate the surgical details and long-term follow-up of a masseter-to-facial nerve transfer in a 3-year-old girl who had insidious onset of a left facial palsy due to a facial nerve schwannoma. After resection, she underwent distal nerve repair with a masseter-to-zygomatic branch transfer. She demonstrated decreased lagophthalmos and good activation and excursion on the left side with near symmetry to the right side, but lacked left frontalis function.

The video can be found here: https://stream.cadmore.media/r10.3171/2022.9.FOCVID22107

## Transcript

We present an operative video demonstrating the transfer of the trigeminal nerve to the facial nerve for facial reanimation.^1–5^

### 0:28 Clinical Presentation.

The patient is a 3-year-old girl who presented with near-complete left facial paralysis. Her parents noted normal facial function at birth, but with progressive decrease in movement over 6 months. Clinically, she had good oral commissure closure without difficulty of oral continence presumed due to reinnervation over the orbicularis oris from the contralateral facial nerve. Additionally, she had trace facial function with moderate difficulty with eyelid closure, but only minimal scleral show due to minimal lagophthalmos at rest.

### 0:58 Imaging Findings.

An MRI of the brain at the time demonstrated an enhancing mass within the petrous bone from the IAC to the stylomastoid foramen. CT of the temporal bone demonstrated enlargement of the facial canal throughout its course.

### 1:10 Resection of Facial Nerve Schwannoma.

The patient underwent a craniotomy and petrosectomy with preservation of the hearing apparatus for resection of a facial nerve schwannoma. After removal of the facial nerve, reconstruction was performed with a 4.5-cm greater auricular nerve graft.

### 1:24 Neurological Examination.

Following tumor removal and nerve grafting surgery, the patient’s facial palsy had mild decrease with worsening lagophthalmos but preserved oral continence.

### 2:05 Rationale for Procedure and Surgical Plan.

With the prolonged denervation time, long segment of grafting, and known outcomes from facial nerve grafting, the patient’s parents understood and wished to pursue smile restoration with a masseter branch transfer to the zygomatic branch of the facial nerve. Proximal grafting for facial nerve injuries often yields unsatisfactory clinical results due to the aberrant targeting from the graft site, as well as co-contraction that often results in unwanted resting tone, or facial synkinesis, whereby muscles unwanted to be activated are activated at the same time, such as closure of the eye and buckling of the mouth. Facial nerve transfers, alternatively, can provide greater strength, which would be as noticeable by greater excursion of the muscle, as well as more isolated movements on attempted smile or other activities. Similarly, if you’re performing a nerve transfer after a nerve graft, it would eliminate some of the potential for synkinesis by removing a pathway for aberrant regeneration. The risks of the surgery would include a failure of the nerve transfer to successfully reinnervate the muscle desired; potential weakness of jaw closure from the donor nerve from the masseter; nuanced synkinesis, particularly involving chewing where the masseter would be activated and the mouth would open; wound healing; and general anesthesia. Surgical alternatives would include cross-facial nerve grafting, temporalis tendon transfer, or continued observation for potential recovery through the graft, and possible free functioning muscle transfer at a later date should the graft not sufficiently recover a satisfactory smile.

### 3:05 Patient Positioning.

The patient was positioned with a head turn toward the right, creating a plane of dissection underneath the submuscular aponeurotic layer of the face. A skin incision was planned to start above the tragus and extended posterior to the mandible. The patient’s prior craniotomy incision is visible, including the extension for obtaining the greater auricular nerve graft.

### 3:23 Surgical Steps.

After injection of epinephrine, a skin incision was made with a 15 blade into the superficial parotid space. Dissection follows superficial to the parotid, while identifying the submuscular aponeurotic layer, where the facial muscles are located. This plane can be very thin in pediatric patients, particularly with chronic denervation. Once this plane is dissected, the facial nerve can be identified, and stimulation confirmed the obvious, that the facial nerve would not stimulate any muscles. The masseter nerve is just deep to the submuscular aponeurotic plane. Once the crural fascia of the masseter is opened, handheld nerve stimulator can be used to identify the branches of the nerve within the masseter muscle. The branches of the trigeminal nerve are traced rostrally within the masseter muscle to identify two primary pedicles. The more superficial masseter nerve was used as a nerve donor to the zygomatic branch of the facial nerve. The donor nerve was cut distally as possible as it started ramifying within the muscle belly. Attention was turned toward the zygomatic branch of the facial nerve, which was further dissected and cleaned of adjacent soft tissue. The ends of the masseter branch and the facial nerves are trimmed to an appropriate length and then suture coapted using 10-0 nylon microsuture. Generally, three interrupted sutures are sufficient to prevent twisting or movement of the suture coaptation for nerves this small in cross-sectional area. The operative field was washed in irrigation, and a small amount of fibrin glue was placed on top of the suture coaptation. The skin was then closed with deep absorbable sutures, as well as interrupted sutures by our plastics colleagues.

A platinum eyelid weight was then placed in the superficial soft tissues of the eyelid. The midline of the eyelid is inspected to ensure appropriate location. Once the skin incision was made, soft tissues were dissected down through the eyelid and superficial to the superior tarsal plate. A linked platinum weight was utilized to allow confirmation to the patient’s globe. Once affixed to the underlying fascial structures, the skin incision was closed with a running, absorbable suture.

### 6:20 Clinical Outcome.

The patient was discharged postoperative day 1. The patient was then seen serially over the next 2 years, which demonstrated improvement in dynamic and static tone. Six-month follow-up demonstrated the start of excursion on the left side of the face with a little bit of contraction. The patient had improved with lagoptholmos, with the use of the eyelid weight, but still had not developed good activation of the left side of the zygomatic branch. Activation of procerus is full. At the 1-year follow-up, improved activation of the zygomatic branch on the left side, nearly similar in excursion to the right. Good oral continence, and good frontalis on the patient’s right side, but not on the left, and good procerus bilaterally. The lack of frontal side activation suggests the graft was not successful at this point in reinnervating frontalis. At the 2-year follow-up, again, she demonstrated a good activation and excursion on the left side, nearly symmetric to the right side with good procerus function, but still lacking frontalis on the left side. Lack of activation of the left-side frontalis indicates that the graft repair of the facial nerve was insufficient to fully reanimate the left side of the face.

Thank you for your interest in this video.

## Acknowledgments

We thank Vance Mortimer for preparation of the video and Kristin Kraus for editorial assistance.

## Disclosures

Dr. Agarwal: consultant for Don Joy Orthopedics. Dr. Mahan: personal fees from joimax, SPR Therapeutics, and Richard Wolf Spine; and grants from Boston Scientific, NIH, University of Utah, and the Neurosurgery Research and Education Foundation, outside the submitted work.

## Author Contributions

Primary surgeon: Mahan, Agarwal. Assistant surgeon: Karsy, Gociman. Editing and drafting the video and abstract: Mahan, Abou-Al-Shaar, Eli. Critically revising the work: Mahan, Abou-Al-Shaar, Karsy. Reviewed submitted version of the work: all authors. Approved the final version of the work on behalf of all authors: Mahan. Supervision: Mahan, Agarwal.
